# Effects of *Klebsiella pneumoniae* Bacteriophages on IRAK3 Knockdown/Knockout THP-1 Monocyte Cell Lines

**DOI:** 10.3390/v14112582

**Published:** 2022-11-21

**Authors:** Bryce Dylan Schubert, Heng Ku, Mwila Kabwe, Trang Hong Nguyen, Helen Irving, Joseph Tucci

**Affiliations:** 1 Department of Rural Clinical Sciences, La Trobe Rural Health School, La Trobe University, P.O. Box 199, Bendigo, VIC 3550, Australia; 2 Commonwealth Scientific and Industrial Research Organisation, Oceans & Atmosphere, Ecosciences Precinct, Dutton Park, QLD 4102, Australia; 3 La Trobe Institute for Molecular Science, La Trobe University, P.O. Box 199, Bendigo, VIC 3550, Australia

**Keywords:** bacteriophages, *Klebsiella pneumoniae*, THP-1 monocytes, IRAK3, cytokines, immune modulation

## Abstract

Bacterial sepsis characterised by an immunosuppressive and cytokine storm state is a challenge to treat clinically. While conventional antibiotics have been associated with exacerbating the cytokine storm, the role that bacteriophages may play in immune modulation of sepsis remains unclear. Bacteriophages are bacterial viruses that have the capacity to lyse specific bacteria and hence provide a natural alternative to antibiotics. *K. pneumoniae* is known to cause sepsis in humans, and in this study we isolated two lytic bacteriophages against this pathogen, one of which was a novel jumbo bacteriophage. We employed THP-1 monocyte cell lines, with different functional phenotypes for the interleukin-1 receptor associated kinase 3 (IRAK3- a cytoplasmic homeostatic mediator and prognostic marker of inflammation), to evaluate the role of the *K. pneumoniae* bacteriophages in modulating the immune response in-vitro. We showed for the first time that bacteriophages did not stimulate excessive production of tumour necrosis factor alpha, or interleukin-6, in THP-1 monocyte cell lines which displayed varying levels of IRAK3 expression.

## 1. Introduction

*Klebsiella pneumoniae* is a Gram-negative rod belonging to the family *Enterobacteriaceae* [1] and a major cause of sepsis and associated mortality [2,3,4]. Sepsis is often characterised by uncontrolled inflammatory responses involving the production of a ‘cytokine storm’ and immune suppression [5]. The cytokine storm is an important response to the bacteria infection. However, during the immune suppressive phase of the response, bacteria are not readily eliminated from the body leading to positive feedback on the ‘cytokine storm’ [6]. Excessive cytokine production is then responsible for multi-organ failure and septic shock, potentially leading to death [5,6].

The exact pathogenesis of sepsis and septic shock is still unclear. Analyses of differentially expressed genes in sepsis have shown that the Interleukin-1 receptor associated kinase 3 (IRAK3) is among the genes whose expression is increased in Toll-like receptor (TLR) signalling pathways [7]. TLR signalling pathways sense pathogen associated molecular patterns (PAMPs) and trigger an innate inflammatory response to eradicate invading microbes [8]. Together with IRAK1, IRAK2 and IRAK4, IRAK3 belongs to a family of cytoplasmic regulators of inflammation [9]. Unlike other IRAK members, IRAK3 downregulates the release of cytokines such as interleukin 6 (IL-6) and tumour necrosis factor alpha (TNF-α) [10]. IRAK3 is also known as IRAK–M as it is mainly found in monocytes and macrophages [10,11]. In sepsis, its activation is implicated in the immune suppressive phase [12].

While elimination of bacteria causing sepsis remains fundamental for its treatment, therapies for sepsis can be problematic [13]. Specifically, bacterial resistance can reduce the efficacy of antibiotics in such applications [2,3,4] and when the bacteria are susceptible, there can be an exacerbation of inflammation in sepsis [14]. This is stimulated by the release of reactive oxygen species, lipopolysaccharide (LPS) and outer membrane vesicles. These in turn promote the release of cytokines as part of the innate immune response [15,16]. It has been suggested, therefore, that the use of antibiotics may create a clinical treatment dilemma between elimination of bacteria and risk of septic shock [17].

Bacteriophages are bacterial viruses that have the capacity to lyse specific bacteria and hence provide a natural alternative to antibiotics. They are bacterial predators that have co-evolved with their prey and are equipped with the capacity to negate some of the bacterial resistance through mechanisms developed in an evolutionary arms race over millennia [18]. Bacteriophages have been shown to be safe when injected in sepsis models in mammals and have not been implicated in excessive cytokine production [19]. In humans, injections of bacteriophages have not been associated with significant adverse events [20,21,22]. It is known that physiological levels of TNF-α and IL-6 are significantly increased in sepsis patients compared to healthy individuals [23,24]. However, when applied in therapy, bacteriophages may normalise TNF-α and IL-6 cytokine levels regardless of initial response [25]. IRAK3 has also been shown to modulate TNF-α and IL-6 in bloodstream infections [10,12,26]. Given the capacity for *K. pneumoniae* to cause sepsis infections in humans, in this study we isolated two lytic bacteriophages against this bacteria, one of which was a novel jumbo bacteriophage. Using these, we aimed to further assess the role of bacteriophages in modulating cytokine production (TNF-α and IL-6), by using IRAK3 knockdown, knockout and wildtype THP-1 monocytes as a model.

## 2. Materials and Methods

### 2.1. THP-1 Monocyte Wild Type, IRAK3 Knockdown and IRAK3 Knockout Cell Culture

The cell lines utilised in this study were wild type THP-1 human monocytes, IRAK-3 knockdown THP-1 human monocytes, and IRAK3 knockout THP-1 human monocytes as described previously [27]. THP-1 cells were cultured in RPMI 1640 medium (Gibco -ThermoFisher Scientific, Scoresby, Australia) with 10% (*v*/*v*) foetal bovine serum (FBS; Gibco -ThermoFisher Scientific, Scoresby, Australia) in 5% CO_2_ at 37 °C. The cells were grown to 70–80% confluence before being passaged, approximately 80% of the volume of cell medium was removed and replaced with fresh RPMI media. The cells were then split for three passages before they were confirmed to be mycoplasma free using the PCR Mycoplasma Detection Kit (TOKU-E, Singapore).

### 2.2. Bacteriophage Isolation and Purification

To isolate bacteriophages, *K. pneumoniae* strain KLEB009 (NCBI: WGS Project: JAERPY01) was cultured in Tryptone Soy Broth (TSB) and on 1% Tryptone Soy Agar (TSA) (Oxoid—ThermoFisher Scientific, Scoresby, Australia) culture plates before incubation at 37 °C aerobically for 24 h. Lytic bacteriophages for *K. pneumoniae* were screened from wastewater samples collected from Victoria, Australia as described previously [28]. Briefly, 9 mL KLEB009 were grown up to 1 × 10^6^ CFU mL-1 before adding 1 mL of 0.2 µm cellulose acetate filtered wastewater. This enrichment was incubated at 37 °C for 4 days before filtering using 0.2 µm Advantec cellulose acetate filters (MicroAnalytix, Caringbah, Australia) and spotting 10 µL of the filtrate onto a freshly prepared lawn of KLEB009 on TSA. These were then incubated for 24 h at 37 °C and examined for clearance zones or plaques caused by bacteriophages. Wastewater samples from two different sites (Gisborne and Woodend, VIC, Australia) were shown to produce plaques on KLEB009. Isolated bacteriophages vB_KpnP_KPN7 (KPN7) and vB_KpnM_KPN8 (KPN8), were then purified from their single plaques and concentrated as described previously [29]. This provided a “crude” purification KPN7 and KPN8. To remove bacterial cellular debris not limited to broken up cell wall components (lipopolysaccharides) or nucleic acids (DNA and RNA) that may pass through the 0.2 µm cellulose acetate filtration (providing a “highly purified” preparation of KPN7 and KPN8), a 10 mL concentrated bacteriophage suspension (approximately 1 × 10^11^ PFU mL^−1^) was treated with 10 µg/mL of DNase I and RNase A for 30 min at room temperature before incubating at 4 °C in 10% *w/v* polyethylene glycol (PEG) 8000 and 1 mol/L NaCl for 15 min. As described previously [30] precipitated bacteriophage particles were treated with 2% *v/v* Triton^®^ X-100 (Sigma-Aldrich^®^, Macquarie Park, Australia) and washed 3 times in phosphate-buffered saline (PBS; pH 7.4) by centrifugation at 12,000× *g* for 15 min. The precipitation, Triton^®^ X-100 treatment and PBS (pH 7.4) wash steps were repeated 3 times before precipitated bacteriophages were filtered using 0.2µm cellulose acetate filters and resuspended in 5 mL RPMI 1640.

### 2.3. Transmission Electron Microscopy

Transmission electron microscopy (TEM) was performed using 400-mesh formvar and carbon copper grids (ProSciTech, Townsville, Australia) as described previously [28]. Bacteriophage stocks (>10^7^ PFU/mL) were allowed to adsorb to the grid for one min before excess solution was removed using filter paper. The grids were then negatively stained three times with 2% (*w*/*v*) uranyl acetate for 20 s. Excess stain was removed by filter paper and grids were air-dried for 20 min before examination under a JEOL JEM-2100 transmission electron microscope. This was operated at an accelerating voltage of 200 kV and high-resolution digital images were recorded on a Gatan Orius SC200D 1 wide angle camera with Gatan Microscopy Suite and Digital Micrograph (Version 2.32.888.0) imaging software. Virions were measured using ImageJ software [31] (Version 1.8.0_112).

### 2.4. Bacteriophage Whole Genome Sequencing and In Silico Analysis

Bacteriophage DNA was extracted as previously described [29]. Ten mL PBS (pH 7.4) suspensions of *Klebsiella* bacteriophages KPN7 and KPN8 at concentrations of 1 × 10^8^ PFU mL^−1^ were treated with 5 mmol/L of MgCl_2_ (Sigma-Aldrich^®^, Macquarie Park, Australia), 10 µg/mL RNAse A, and 10 µg/mL DNAse I (Promega, Alexandria, Australia) for 30 min at room temperature. Using 10% (*w*/*v*) PEG-8000 and 1 g/L sodium chloride (Sigma-Aldrich^®^, Macquarie Park, Australia), the bacteriophages were then precipitated at 4 °C and resuspended in 50 mL nuclease-free water (Promega, Alexandria, Australia). Viral proteins were then digested with 50 µg/mL of proteinase K, 20 mmol/L EDTA (Sigma-Aldrich^®^, Macquarie Park, Australia) and 0.5% (*v*/*v*) of sodium dodecyl sulfate (Sigma-Aldrich^®^, Macquarie Park, Australia) for 1 h at 55 °C before an equal volume of phenol-chloroform-isoamyl alcohol (29:28:1) (Sigma-Aldrich^®^, Macquarie Park, Australia) was added to the DNA/bacteriophage protein mixture. The DNA and protein were separated by centrifugation at 12,000× *g* for 10 min to collect DNA in the aqueous phase before incubating it with 200 µL of absolute isopropanol-2-propanol (Sigma-Aldrich^®^, Macquarie Park, Australia) at −20 °C overnight. Following centrifugation for 10 min at 12,000× *g*, the supernatant was taken and discarded, and the DNA pellet washed with 200 µL 70% ethanol (ThermoFisher Scientific, Scoresby, Australia) before 2 min centrifugation at 12,000× *g*. The 70% ethanol (ThermoFisher Scientific, Scoresby, Australia) was discarded then the DNA pellet resuspended in 30 µL nuclease-free water (Promega, Alexandria, Australia).

The extracted bacteriophage DNA was then sequenced using the Illumina MiSeq^®^ technology. DNA libraries were prepared using the DNA Library Prep Kit (NEB) and a MiSeq^®^ V3 600 cycle reagent kit (Illumina, Melbourne, Australia) according to manufacturer’s instructions. Generated reads were trimmed using Trim Galore v0.6.4 with the default settings (Q scores of ≥20, with automatic adapter detection) and assembled using the Unicycler de novo assembly pipeline [32]. The bacteriophage genomes were then annotated automatically using the Rapid Annotation Subsystem Technology (RAST) [33] and quality checked manually using Geneious Prime 2022.2.1 (https://www.geneious.com/ (accessed on 10 October 2022)) employing the bacterial translation Table 11 including codon start ATG, TTG, and GTG. To predict taxa, bacteriophage whole genomes were uploaded to the Viral Proteomic Tree (ViPTree) webserver to construct a viral proteomic tree with other related bacterial viral genomes in the reference database [34]. To assess the phylogeny of the isolated bacteriophages, closely related genomes were download from NCBI nucleotide BLAST [35]. Based on the evolutionary relatedness of putative capsid proteins, putative terminase proteins, and putative DNA polymerase proteins where available, phylogenetic trees were constructed using the APE package [36] and annotated using GGTREE package [37] in the programming language R version 2022.07.1+554 (https://www.r-project.org/ (accessed on 10 October 2022)).

### 2.5. Assessment of THP-1 Monocyte In-Vitro Cytokine Production

Wild type and IRAK3 knockout/knockdown THP-1 cells were split at approximately 2 × 10^4^ cells in 300 µL media per well in Corning^®^ Costar cell culture clear flat bottom 96 well plates (Sigma-Aldrich^®^, Macquarie Park, Australia). Cells were treated with 1 × PBS as the negative control, 1 µg/mL lipopolysaccharide (LPS, *Escherichia coli* 055:B5; Merck, Bayswater, Australia) as the positive control, or 3.0 × 10^6^ plaque forming units (PFU) per mL of crudely purified or highly purified (see above) suspensions of bacteriophages KPN7 or KPN8 for 24 h. 100 µL of cell supernatants were then collected and measured for concentrations of human cytokines (TNF-α and IL-6) using BD OptEIA™IL-6 and OptEIA™TNF-α ELISA kits (BD Biosciences, North Ryde, Australia) according to the manufacturer’s instructions.

### 2.6. Statistical Analysis

Experimental data were collected as above to assess whether there were differences in cytokine production between THP-1 monocytes with varying expression levels of IRAK3, and treated with different preparations of bacteriophages, LPS or PBS. Using the Shapiro–Wilk normality test, data was found to be significantly skewed, hence, the difference in the medians of IL-6 and TNF-α production between the treatment groups was compared using the Kruskal–Wallis non-parametric test. All data were visualised as boxplots comprising the five-member statistic median, 25th percentile, 75th percentile and the upper (Q3 + 1.5 × IQR) and the lower limit (Q1 − 1.5 × IQR). Bacteriophage morphology characteristics were normally distributed and summarised as standard error of mean. All statistical analyses were performed in R version 2022.07.1+554 (https://www.r-project.org/ (accessed on 10 October 2022)).

## 3. Results

### 3.1. Characterisation of Klebsiella Bacteriophages KPN7 and KPN8

**Figure 1:**
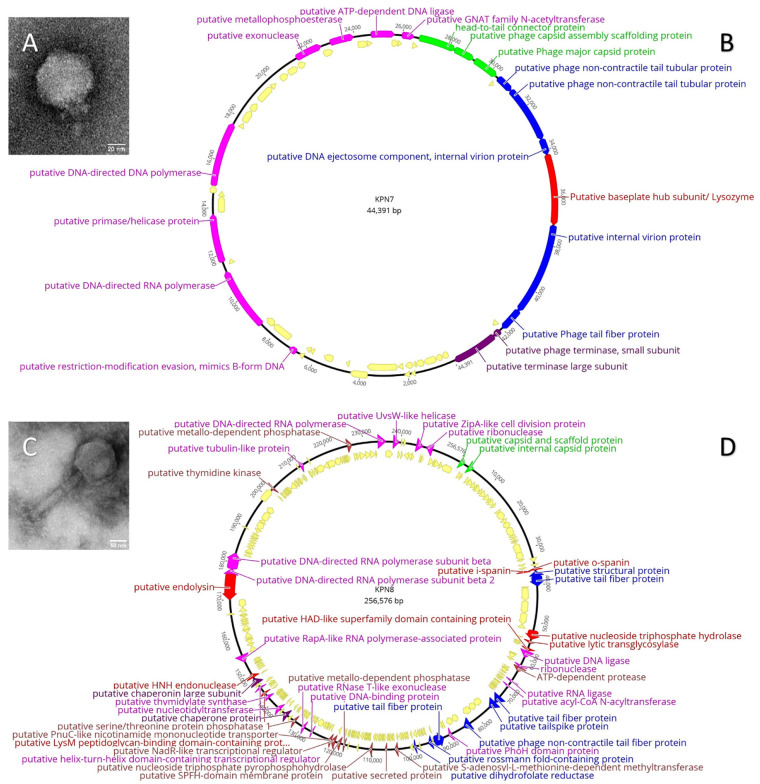
Characterisation of bacteriophages KPN7 and KPN8. (**A**,**C**): Electron micrographs of bacteriophage KPN7 (**A**) (Capsid dimensions: diameter = 62.3 ± 1 nm, Tail length = 15.3 ± 3 nm, Tail width = 15.6 ± 1 nm) showing a typical Podovirus morphology and bacteriophage KPN8 (**C**) (Capsid dimensions: diameter = 130.2 ± 3 nm, Tail length = 196.1 ± 4 nm, Tail width = 53.7 ± 11 nm) shown with a typical Myovirus morphology. (**B**,**D**): Genomic representation of bacteriophages KPN7 (**B**) and KPN8 (**D**), shown in circular form for ease of visualisation. Putative functions of genes are denoted by purple (putative packaging protein), green (putative head or capsid structural protein), blue (putative tail structural genes), red (putative lysin proteins), brown (putative metabolic proteins), and pink (putative DNA manipulation proteins). Hypothetical proteins are shown in yellow.

Two bacteriophages, KPN7 and KPN8, were isolated from samples of wastewater from treatment plants in Gisborne and Woodend, respectively (VIC, Australia). A summary of their characteristics is highlighted in Table 1. An assessment of the TEM revealed that the bacteriophages KPN7 and KPN8 were Podovirus and Myovirus, respectively (Figure 1A,C). Capsid diameters were recorded at mean (SE) 62.3 (1.1) nm for the bacteriophage KPN7 and mean (SE), 130.2 (3.2) nm for the bacteriophage KPN8. The tails lengths were 15.3 (2.7) nm (KPN7) and 196.1 (3.5) nm (KPN8) while tail widths were 15.6 (1.4) nm and 53.7 (11.4) nm for KPN7 and KPN8, respectively.

In silico analysis of the bacteriophage DNA sequences revealed a 44 kb *Autographiviridae*, with GC% content of 52% and 57 open reading frames (ORFs) for the bacteriophage KPN7. Of the 57 annotated ORFs in the bacteriophage KPN7 genome, approximately two-thirds (66.7%) were denoted as hypothetical proteins (Figure 1B). The much larger bacteriophage KPN8 genome (referred to as a Jumbo bacteriophage) contained 257 kb with a GC% content of 44%, and 83.6% (249/298) of the genome annotated as hypothetical proteins (Figure 1D). These annotated genomes have been submitted to GenBank^®^ under accession numbers OP079918 and OP079919 for bacteriophages KPN7 and KPN8, respectively. Further analysis did not reveal the presence of putative tRNAs, tmRNAs [38], CRISPR sequences [39], antibiotic resistance genes [40] or toxins. Annotations are summarised in Tables S1 and S2.

### 3.2. Phylogeny of Klebsiella Bacteriophages KPN7 and KPN8

**Figure 2:**
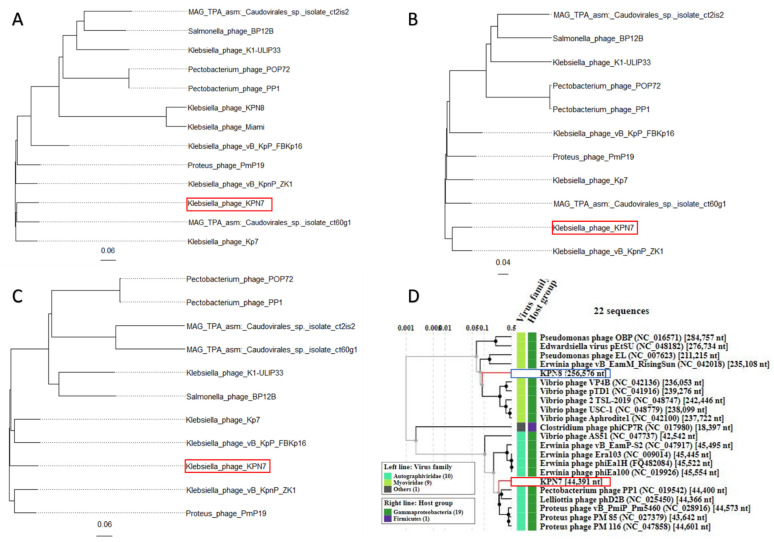
Phylogenetic and proteomic analysis of the bacteriophages KPN7 and KPN8 (highlighted in red and blue rectangles, respectively). Phylogenetic tree construction carried out with 100 bootstraps for putative capsid protein (**A**) putative terminase protein (**B**) and putative DNA polymerase proteins (**C**). To ascertain genomic viral taxa, viral protein analysis was constructed using VIPTree webserver (**D**).

Phylogenetic analysis using the capsid proteins revealed that bacteriophage KPN7 formed a clade with the bacteriophage MAG TPA asm: *Caudovirales* sp. isolate ct60g1 (Accession number: BK056972) (Figure 2A). Using NCBI nucleotide BLAST, a query of the whole genome revealed that bacteriophage KPN7 was most closely related to the *Klebsiella* phage vB_KpnP_ZK1 (accession number: OK625527) (query cover of 94% and identity of 97.3%) with which it formed a monophyletic clade in the phylogenetic analysis of the putative terminase genes (Figure 2B). Phylogenetic analysis using the putative DNA polymerase gene revealed that bacteriophage KPN7 did not form a clade with either of these aforementioned bacteriophages (Figure 2C).

Whole genome NCBI nucleotide BLAST for the bacteriophage KPN8 could not be completed due to its large size. However, many sections of its genome were found to BLAST to the previously reported novel *Klebsiella* jumbo phage Miami (MT701590) [41]. When analysed in a phylogenetic tree of putative capsid proteins, these two jumbo bacteriophages clustered together in a clade (Figure 2A). Unlike the analyses for KPN7, for KPN8, it was not possible to undertake phylogenetic analysis using putative terminase and DNA polymerase genes, as putative genes for these could not be annotated from the KPN8 genome. Viral proteomic analysis revealed that KPN8 was more closely related to the *Myoviridae* family of bacteriophages while KPN7 formed a clade with bacteriophages from the *Autographiviridae* family (Figure 2D).

### 3.3. Effect of KPN7 and KPN8 Bacteriophages on IL-6 and TNF-α Cytokine Production in Wild Type, IRAK3 Knockdown and IRAK3 Knockout THP-1 Monocytes

**Figure 3:**
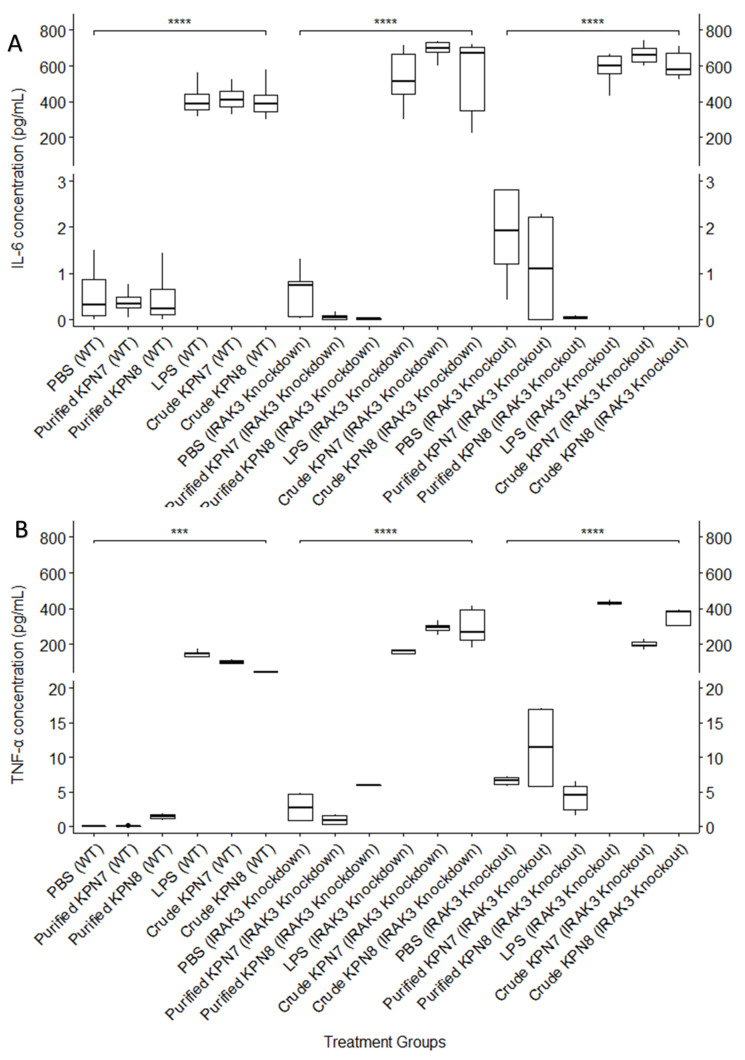
Effects of PBS, LPS (1 µg/mL) and bacteriophage treatments on cytokine production by wild-type (WT), IRAK3 knockdown and IRAK3 knockout THP-1 monocytes. (**A**) Interleukin 6 production (**B**) Tumour necrosis factor alpha production. *** *p* value <0.001; **** *p* value < 0.0001.

KPN7 and KPN8 were tested for their capacity to stimulate the production of IL-6 and TNF-α in monocyte cell lines, including those with an IRAK3 knockdown (partial expression) and a complete IRAK3 knockout (lacking IRAK3 expression) phenotype. In this analysis, the crude bacteriophage preparations involving only the 0.2 µm filtration process (sufficient to remove whole bacterial cells, but not bacterial cell debris), and the highly purified bacteriophage suspensions (where bacterial cell debris such as endotoxin and LPS is removed, as described in the methods) were assessed. Both KPN7 and KPN8 revealed relatively similar results with the highly purified preparations, showing similar levels of IL-6 stimulation as the PBS control. The crude preparations induced a significantly higher IL-6 level in comparison, in all monocyte cells regardless of the IRAK3 functionality (*p* < 0.001) (Figure 3A). The IL-6 levels induced by the crudely prepared bacteriophage suspensions was similar to those induced by commercial LPS solution (Figure 3A). This observation was consistent with that seen in the TNF-α induced assays (Figure 3B). Combining the data from all the six samples tested in each of the three phenotypically distinct monocyte cell lines, it was observed that for both IL-6 and TNF-α stimulation the wild type cells showed the lowest inductions followed by the monocytes with IRAK3 knockdown, and highest levels were seen in the IRAK3 knockout THP-1 monocytes (*p* < 0.001). IL-6 production stimulated by purified KPN7 and KPN8 was lower (less than 2 pg/mL) across all cell lines used. In the wild-type THP-1 monocytes, IL-6 was at a mean (Q1–Q3) of 0.3 (0.2–0.5) pg/mL and 0.2 (0.1–0.7) pg/mL for KPN7 and KPN8, respectively. In the IRAK3 knockdown THP-1 monocytes, the IL-6 mean (Q1–Q3) concentrations for KPN7 and KPN8 were 0.04 (0.007–0.09) pg/mL and 0.01 (0.007–0.03) pg/mL, while in the IRAK3 knockout cells, the mean (Q1–Q3) was 1.1 (0.01–2.2) and 0.05 (0.03–0.08), for KPN7 and KPN8, respectively. Similarly induced TNF-α were also lower. Mean (Q1–Q3) of the TNF-α levels for the purified KPN7 and KPN8 in the wild-type THP-1 monocyte cell lines were 0.02 (0.01–0.06) pg/mL and 1.44 (1.1–1.67) pg/mL, respectively. In the IRAK3 knockdown THP-1 monocytes, the mean (Q1–Q3) TNF-α levels induced by KPN7 were 0.93 (0.26–1.61) pg/mL while those produced by the KPN8 were 5.93 (5.87–5.98) pg/mL. When monocytes had no functional IRAK3, the purified bacteriophages stimulated mean (Q1–Q3) TNF-α levels that were 11.37 (5.8–16.95) pg/mL and 4.56 (2.4–5.78) pg/mL for KPN7 and KPN8, respectively. The data on the mean and quantiles across all cell lines are summarised in supplementary Tables S3 and S4.

## 4. Discussion

This study isolated and characterised two bacteriophages targeting the *K. pneumoniae* bacteria strain KLEB009 (NCBI: WGS Project: JAERPY01). The GC% content of both bacteriophages KPN7 and KPN8 were lower (51% and 44%, respectively) than the 57.5% of the host KLEB009. Bacteriophage GC% content has been known to be close to that of their host, and in cases where they have lower GC% content than their host, it has been suggested that bacteriophages may carry tRNAs to complement potential biochemical shortfalls [42,43]. In-silico analysis of both KPN7 and KPN8, revealed no tRNAs or tmRNAs. No putative antibiotic resistance genes, CRISPR sequences, integrase genes or toxins were identified in the genomes of KPN7 or KPN8, and so these two bacteriophages may possibly be candidates for use in bacteriophage therapy. Phylogenetic analyses using the terminase gene revealed that KPN7 formed a monophyletic clade with *Klebsiella* phage vB_KpnP_ZK1 (accession number: OK625527), and with the bacteriophage MAG TPA_asm: *Caudovirales* sp. isolate ct60g1 (Accession number: BK056972), when using the capsid proteins. Phylogenetic analysis using the capsid protein showed that KPN8 differed markedly from most of the bacteriophages with which it clustered, and formed a monophyletic clade with the novel *Klebsiella* phage Miami, another Jumbo bacteriophage.

We demonstrated that introducing purified *K. pneumoniae* bacteriophages into cultures of wild type THP-1 monocytes had little effect; levels of TNF-α and IL-6 cytokines were similar to the low levels produced by negative control PBS. Other studies using monocytes and purified bacteriophages showed similarly low levels of cytokines in their models [44]. The levels of TNF-α and IL-6 stimulated by the purified bacteriophages in the IRAK3 knockdown monocytes were also similar to those produced by the negative control PBS. In the wild-type and IRAK3 knockdown THP-1 monocytes (Figure 3), the addition of KPN7 and KPN8 resulted in concentrations of the measured cytokines that fell within the known physiological range in humans (<2 pg/mL IL-6 and 5.5 pg/mL TNF-α, respectively) [23,45]. In the IRAK3 knockout THP-1 monocytes, the TNF-α levels were above the physiological range for both PBS and KPN7. There was no statistical difference between PBS and KPN7 TNF-α concentration in the IRAK3 knockout THP-1 monocytes.

The tests using crudely prepared bacteriophage suspensions highlight the importance of removing endotoxin and other bacterial cell debris to prevent immune stimulation. That the purified bacteriophages did not elicit an extreme immune response may be due to the fact that these viruses do not target cells of higher organisms, and that humans have had extensive exposure to them (including regular presence in the circulation, from gut origins) throughout their evolution [46]. Others have shown that regular bacteriophage injections into human subjects including those with immunodeficiency, does not stimulate extensive responses or adverse effects [47].

The low levels of TNF-α and IL-6 in both the wild type and the IRAK3 knockdown THP-1 monocytes suggest that the pure bacteriophages are not recognised by PRRs such as TLRs on human monocytes. Studies exploring this relationship are very limited, however, one study suggests that T4 bacteriophage (lytic for *Escherichia coli*) failed to induce expression of TLR2 and TLR4 in human monocytes [48]. Therefore, the downstream immune response was not induced, and cytokine levels remained minimal or unchanged. Bacteriophages did not cause an inflammatory response in some studies [49,50,51], while others have suggested their increased presence is linked to aggravated intestinal inflammation, which could also be attributed to a microbiome shift [52]. Research that further explores these issues would greatly enhance the understanding of bacteriophage and immune receptor dynamics. This notwithstanding, the work presented here, and that of others showing similar minimal stimulation of immune cells, suggests that bacteriophages may present a relatively safe option, if needed to be delivered internally for therapy.

The IRAK3 knockdown and knockout THP-1 monocytes were included in this study as a model to evaluate if individuals with dysregulated immune response have increases in inflammatory cytokines induced by bacteriophages. To our knowledge, this is the first study to assess the effects of these viruses on monocytes where the negative attenuation of IRAK3 is diminished or abolished. What was interesting in this investigation was that there was little difference when the purified bacteriophage was added to the IRAK3 knockdown THP-1 monocytes or the wild type monocytes. Despite the reduced levels of IRAK3 in the knockdown monocyte cell line and an absence of detectable IRAK3 protein in the knockout monocyte cell line [27], the purified bacteriophage did not induce a dramatic increase in secretion of TNF-α and IL-6 cytokines. Predictably, in the IRAK3 knockdown and wild type THP-1 monocytes, the unpurified bacteriophages elicited high concentrations of the cytokines due to the presence of LPS and other bacterial remnants. In the purified bacteriophage assays, we hypothesised that there would be a reduced output of TNF-α and IL-6 based on the removal of LPS, and the results confirmed this.

Therefore, our work has verified that some form of endotoxin removal needs to occur before application of bacteriophages clinically or in veterinary applications. Bacteriophage therapy is being applied clinically in humans in isolated cases, especially on compassionate grounds, where antibiotics and other treatments have failed [53,54]. In these cases, they have shown efficacy. Further testing in animal models and clinical trials is necessary for regulatory bodies to accept that this innovative approach to antimicrobial therapy is viable.

## Figures and Tables

**Table 1:** KPN7 and KPN8 morphological and genomic characteristics.

	Characteristics	KPN7	KPN8
TEM	Morphology	Podovirus	Myovirus
	Capsid diameter, mean (SE)	62.3 (1.1) nm	130.2 (3.2) nm
	Tail length, mean (SE)	15.3 (2.7) nm	196.1 (3.5) nm
	Tail width, mean (SE)	15.6 (1.4) nm	53.7 (11.4) nm
Genomics	Genome size	44 391 bases	256 576 bases
	GC% content	51.80%	44.10%
	Hypothetical genes, *n* (%)	38/57 (66.7%)	249/298 (83.6%)
	Putative viral family	*Autographiviridae*	*Myoviridae*

## Data Availability

Genomic sequences for the bacteriophages KPN7 and KPN8 are submitted to NCBI GenBank under accession numbers OP079918 and OP079919, respectively. All other data is presented here and available in supplementary data.

## Supplementary Materials

The following supporting information can be downloaded at: https://www.mdpi.com/article/10.3390/v14112582/s1, Table S1: Bacteriophage KPN7 annotations; Table S2: Bacteriophage KPN8 annotations; Table S3: Production of interleukin 6; Table S4: Production of Tumour Necrosis Factor—alpha.
