# Supplementary material for: Effects of Klebsiella pneumoniae Bacteriophages on IRAK3 Knockdown/Knockout THP-1 Monocyte Cell Lines
Source: Viruses. 2022 Nov 21;14(11):2582. doi: 10.3390/v14112582 (PMC9699088; doi:10.3390/v14112582)
Supplement: Supplementary file 1 [file viruses-14-02582-s001.zip › viruses-1990293-supplementary.pdf]

Effects of Klebsiella pneumoniae Bacteriophages on IRAK3 Knockdown/Knockout THP-1 Monocyte Cell Lines

Bryce Dylan Schubert 1,†, Heng Ku 2,†, Mwila Kabwe 1,3,†, Trang Hong Nguyen 1,3, Helen Irving 1,3 and Joseph Tucci 1,3,*

1 Department of Rural Clinical Sciences, La Trobe Rural Health School, La Trobe University, P.O. Box 199, Bendigo, VIC 3550, Australia

2 Commonwealth Scientific and Industrial Research Organisation, Brisbane, QLD, Australia

3 La Trobe Institute for Molecular Science, La Trobe University, P.O. Box 199, Bendigo, VIC 3550, Australia

* Correspondence: j.tucci@latrobe.edu.au

† These authors contributed equally to this work.

Supplementary Tables

Table S1: Bacteriophage KPN7 annotations

Name	Start	Finish	Length
putative terminase large subunit	42493	44391	1899
putative phage terminase, small subunit	42152	42493	342
hypothetical protein	41974	42204	231
putative Phage tail fiber protein	41018	42007	990
putative internal virion protein	37182	41021	3840
Putative baseplate hub subunit/ Lysozyme	34203	37139	2937
putative DNA ejectosome component, internal virion protein	33534	34202	669
putative phage non-contractile tail tubular protein	31138	33534	2397
putative phage non-contractile tail tubular protein	30410	31150	741
hypothetical protein	30301	30441	141
putative Phage major capsid protein	29182	30273	1092
putative phage capsid assembly scaffolding protein	28255	29106	852
head-to-tail connector protein	26687	28255	1569
hypothetical protein	26479	26685	207
putative GNAT family N-acetyltransferase	25979	26470	492
hypothetical protein	25836	26012	177
hypothetical protein	25577	25852	276
putative ATP-dependent DNA ligase	24697	25668	972
hypothetical protein	24506	24700	195
hypothetical protein	24078	24509	432
putative metallophosphoesterase	22954	23961	1008
putative HNH endonuclease motif	22552	22953	402
putative exonuclease	21440	22567	1128
hypothetical protein	21327	21650	324
hypothetical protein	20806	21258	453
hypothetical protein	20375	20746	372
hypothetical protein	19866	20084	219
hypothetical protein	19060	19866	807
hypothetical protein	18508	18885	378
hypothetical protein	17923	18456	534
hypothetical protein	17630	17851	222
putative DNA-directed DNA polymerase	14934	17618	2685
hypothetical protein	14628	14927	300
hypothetical protein	14495	14722	228
hypothetical protein	13794	14498	705
putative primase/helicase protein	11712	13730	2019
hypothetical protein	11539	11727	189
putative DNA-directed RNA polymerase	8661	11297	2637
hypothetical protein	8282	8659	378
hypothetical protein	7351	8253	903
putative restriction-modification evasion, mimics B-form DNA	6817	7152	336
hypothetical protein	6469	6657	189

hypothetical protein	6305	6505	201
hypothetical protein	6153	6308	156
hypothetical protein	5271	5678	408
hypothetical protein	4528	4653	126
hypothetical protein	3689	4411	723
hypothetical protein	2442	3722	1281
hypothetical protein	2179	2397	219
hypothetical protein	1931	2146	216
hypothetical protein	1777	2052	276
hypothetical protein	1698	1862	165
hypothetical protein	1597	1719	123
putative endolysin	1257	1604	348
hypothetical protein	708	1244	537
hypothetical protein	431	697	267
hypothetical protein	151	417	267

Table S2: Bacteriophage KPN8 annotations

Name	Start	Finish	Length
hypothetical protein	252695	255610	2916
hypothetical protein	251973	252653	681
hypothetical protein	250471	251976	1506
hypothetical protein	250080	250478	399
hypothetical protein	248745	250070	1326
putative ribonuclease	246945	248666	1722
hypothetical protein	246319	246900	582
hypothetical protein	245909	246313	405
putative ZipA-like cell division protein	244260	245876	1617
hypothetical protein	243502	244242	741
hypothetical protein	242607	243491	885
hypothetical protein	241322	242614	1293
hypothetical protein	240775	241266	492
hypothetical protein	240449	240820	372
hypothetical protein	240057	240455	399
hypothetical protein	239573	240079	507
putative UvsW-like helicase	237987	239537	1551
hypothetical protein	235991	237970	1980
putative DNA-directed RNA polymerase	233896	236007	2112
hypothetical protein	233112	233462	351
hypothetical protein	231664	233055	1392
hypothetical protein	230176	231648	1473
hypothetical protein	228556	230142	1587
hypothetical protein	227694	228437	744
hypothetical protein	226853	227653	801
putative metallo-dependent phosphatase	225714	226853	1140
hypothetical protein	224959	225720	762
hypothetical protein	224549	224956	408
hypothetical protein	224368	224493	126
hypothetical protein	223928	224290	363
hypothetical protein	223524	223928	405
hypothetical protein	222882	223517	636
hypothetical protein	221276	222895	1620
hypothetical protein	219192	221204	2013
hypothetical protein	217821	218933	1113
hypothetical protein	215673	217775	2103

hypothetical protein	215158	215571	414
hypothetical protein	214844	215161	318
hypothetical protein	214525	214872	348
hypothetical protein	214179	214547	369
hypothetical protein	213322	213891	570
hypothetical protein	213000	213305	306
putative tubulin-like protein	211923	212885	963
hypothetical protein	211401	211589	189
hypothetical protein	211026	211331	306
hypothetical protein	210836	211060	225
hypothetical protein	209686	210420	735
hypothetical protein	209525	209641	117
hypothetical protein	209193	209345	153
hypothetical protein	208618	208815	198
hypothetical protein	207682	208542	861
hypothetical protein	206183	207673	1491
hypothetical protein	205634	206125	492
hypothetical protein	205119	205625	507
hypothetical protein	204958	205113	156
hypothetical protein	204792	204938	147
hypothetical protein	204364	204795	432
hypothetical protein	204050	204361	312
hypothetical protein	203373	204053	681
hypothetical protein	202927	203370	444
putative thymidine kinase	202251	202856	606
hypothetical protein	201990	202205	216
hypothetical protein	198365	201928	3564
hypothetical protein	196731	198446	1716
hypothetical protein	194135	196738	2604
hypothetical protein	193251	194129	879
hypothetical protein	190933	193092	2160
hypothetical protein	190021	190917	897
hypothetical protein	189767	189961	195
hypothetical protein	189312	189806	495
hypothetical protein	188553	189254	702
hypothetical protein	188173	188451	279
hypothetical protein	187659	188102	444
hypothetical protein	186878	187528	651
hypothetical protein	185928	186791	864
hypothetical protein	185188	185913	726
hypothetical protein	183673	185172	1500
hypothetical protein	183509	183670	162
hypothetical protein	182592	183509	918
putative DNA-directed RNA polymerase subunit beta	178391	182647	4257
putative DNA-directed RNA polymerase subunit beta 2	176781	178394	1614
putative endolysin	169954	176760	6807
hypothetical protein	167761	169911	2151
hypothetical protein	166592	167710	1119
hypotehetical protein	166091	166519	429
hypothetical protein	165526	166119	594
hypothetical protein	164961	165455	495
hypothetical protein	164231	164929	699
hypothetical protein	163793	164221	429
hypothetical protein	163116	163790	675
hypothetical protein	162687	163109	423
hypothetical protein	161231	162685	1455

hypothetical protein	160374	161171	798
hypothetical protein	158833	160323	1491
hypothetical protein	158353	158766	414
hypothetical protein	156995	158332	1338
hypothetical protein	156325	156984	660
hypothetical protein	154978	156255	1278
putative RapA-like RNA polymerase-associated protein	152899	154953	2055
hypothetical protein	152305	152847	543
hypothetical protein	151724	152296	573
hypothetical protein	151411	151710	300
hypothetical protein	151198	151404	207
hypothetical protein	150616	151092	477
hypothetical protein	150012	150572	561
putative thymidine kinase	149373	149999	627
putative HNH endonuclease	148330	149412	1083
hypothetical protein	148054	148311	258
putative chaperonin large subunit	146434	148011	1578
hypothetical protein	145810	146343	534
hypothetical protein	145344	145901	558
putative helix-turn-helix domain-containing transcriptional regulator	144908	145282	375
hypothetical protein	144294	144818	525
hypothetical protein	143936	144313	378
hypothetical protein	143508	143924	417
putative LysM peptidoglycan-binding domain-containing protein	143010	143492	483
hypothetical protein	142507	142887	381
putative thymidylate synthase	141585	142448	864
hypothetical protein	141135	141572	438
hypothetical protein	140775	141131	357
hypothetical protein	140593	140784	192
hypothetical protein	140192	140599	408
hypothetical protein	139733	140182	450
hypothetical protein	139367	139705	339
hypothetical protein	139078	139290	213
hypothetical protein	138815	139081	267
hypothetical protein	138435	138722	288
hypothetical protein	138250	138438	189
putative nucleotidyltransferase	137261	138253	993
hypothetical protein	137016	137264	249
hypothetical protein	136438	136899	462
hypothetical protein	135936	136490	555
putative chaperone protein	134688	135911	1224
hypothetical protein	134241	134678	438
hypothetical protein	133587	134231	645
hypothetical protein	133262	133636	375
hypothetical protein	132909	133265	357
putative serine/threonine protein phosphatase 1	132115	132912	798
hypothetical protein	131683	132090	408
hypothetical protein	131231	131659	429
hypothetical protein	130742	131170	429
hypothetical protein	130176	130745	570
hypothetical protein	129862	130182	321
putative RNase T-like exonuclease	129155	129829	675
hypothetical protein	128399	129142	744
hypothetical protein	127955	128374	420
hypothetical protein	127387	127965	579
hypothetical protein	127051	127332	282

putative DNA-binding protein	126800	126976	177
hypothetical protein	125087	126799	1713
hypothetical protein	124276	125061	786
hypothetical protein	123758	124276	519
hypothetical protein	123445	123747	303
hypothetical protein	122814	123329	516
hypothetical protein	122013	122714	702
putative PnuC-like nicotinamide mononucleotide transporter	121292	121987	696
putative NadR-like transcriptional regulator	120182	121291	1110
hypothetical protein	119553	120167	615
putative SPFH-domain membrane protein	118674	119540	867
hypothetical protein	118333	118677	345
hypothetical protein	117996	118256	261
putative metallo-dependent phosphatase	117460	117993	534
hypothetical protein	117134	117460	327
hypothetical protein	116800	117132	333
hypothetical protein	116455	116763	309
hypothetical protein	116207	116518	312
hypothetical protein	115916	116185	270
hypothetical protein	115674	115919	246
hypothetical protein	115204	115623	420
hypothetical protein	114620	115207	588
hypothetical protein	114163	114549	387
hypothetical protein	113850	114290	441
hypothetical protein	113012	113875	864
hypothetical protein	112466	112918	453
hypothetical protein	112203	112451	249
hypothetical protein	111781	112203	423
hypothetical protein	111502	111750	249
hypothetical protein	111253	111477	225
hypothetical protein	111041	111250	210
hypothetical protein	110769	111044	276
putative nucleoside triphosphate pyrophosphohydrolase	110105	110722	618
hypothetical protein	109535	110086	552
hypothetical protein	108894	109538	645
hypothetical protein	108414	108818	405
hypothetical protein	107853	108344	492
hypothetical protein	107412	107807	396
hypothetical protein	107072	107425	354
hypothetical protein	106711	107058	348
putative secreted protein	106238	106696	459
hypothetical protein	105831	106208	378
hypothetical protein	105412	105804	393
hypothetical protein	104926	105402	477
hypothetical protein	104569	104883	315
hypothetical protein	104101	104556	456
putative S-adenosyl-L-methionine-dependent methyltransferase	103378	104073	696
hypothetical protein	102952	103368	417
hypothetical protein	101881	102756	876
hypothetical protein	101430	101795	366
hypothetical protein	100875	101402	528
hypothetical protein	100416	100850	435
hypothetical protein	99984	100391	408
hypothetical protein	99254	99928	675
hypothetical protein	98911	99354	444
putative dihydrofolate reductase	98388	98909	522

hypothetical protein	97537	98391	855
hypothetical protein	97264	97518	255
hypothetical protein	96756	97304	549
hypothetical protein	96260	96763	504
hypothetical protein	95928	96233	306
hypothetical protein	95723	95941	219
hypothetical protein	95164	95730	567
hypothetical protein	94963	95151	189
putative rossmann fold-containing protein	93989	94912	924
hypothetical protein	93539	93937	399
hypothetical protein	93339	93614	276
putative tail fiber protein	90655	93225	2571
hypothetical protein	89811	90608	798
hypothetical protein	89243	89710	468
putative PhoH domain protein	88379	89152	774
hypothetical protein	86542	88311	1770
hypothetical protein	84750	86480	1731
putative phage non-contractile tail fiber protein	82822	84711	1890
hypothetical protein	80555	82696	2142
hypothetical protein	78681	80441	1761
hypothetical protein	76611	78665	2055
putative tailspike protein	74158	76299	2142
putative tail fiber protein	72196	74076	1881
hypothetical protein	71693	72046	354
hypothetical protein	71134	71661	528
hypothetical protein	70716	71102	387
hypothetical protein	70420	70734	315
hypothetical protein	70049	70423	375
hypothetical protein	69297	70052	756
hypothetical protein	68871	69251	381
putative acyl-CoA N-acyltransferase	68379	68846	468
hypothetical protein	68098	68379	282
hypothetical protein	67709	68029	321
hypothetical protein	67239	67667	429
putative RNA ligase	66758	67225	468
hypothetical protein	66645	66761	117
hypothetical protein	65933	66268	336
hypothetical protein	65821	65925	105
hypothetical protein	64939	65817	879
hypothetical protein	64613	64849	237
hypothetical protein	64040	64534	495
ATP-dependent protease	62636	63976	1341
ribonuclease	62112	62633	522
hypothetical protein	61454	62059	606
hypothetical protein	60158	61450	1293
hypothetical protein	59831	60157	327
putative DNA ligase	57916	59820	1905
putative HAD-like superfamily domain containing protein	57211	57837	627
hypothetical protein	56732	57202	471
hypothetical protein	56302	56712	411
hypothetical protein	55985	56242	258
putative lytic transglycosylase	55226	55894	669
hypothetical protein	54851	55135	285
putative nucleoside triphosphate hydrolase	52282	54846	2565
hypothetical protein	51335	52321	987
hypothetical protein	48072	51287	3216

hypothetical protein	44514	48026	3513
hypothetical protein	40819	44466	3648
hypothetical protein	40486	40749	264
putative tail fiber protein	37669	40476	2808
putative structural protein	36371	37633	1263
putative i-spanin	35967	36359	393
putative o-spanin	35567	36100	534
hypothetical protein	35020	35514	495
hypothetical protein	34518	35117	600
hypothetical protein	33895	34542	648
hypothetical protein	32900	33883	984
hypothetical protein	32559	32966	408
hypothetical protein	30465	32552	2088
hypothetical protein	27470	30439	2970
hypothetical protein	27016	27411	396
hypothetical protein	26555	26947	393
hypothetical protein	25919	26491	573
hypothetical protein	25141	25776	636
hypothetical protein	22639	25032	2394
hypothetical protein	20972	22642	1671
hypothetical protein	19938	20960	1023
hypothetical protein	17572	19758	2187
hypothetical protein	16926	17483	558
hypothetical protein	14809	15315	507
hypothetical protein	13423	14769	1347
hypothetical protein	12764	13426	663
hypothetical protein	11466	12761	1296
hypothetical protein	9948	11462	1515
hypothetical protein	9090	9878	789
hypothetical protein	7743	9083	1341
hypothetical protein	7346	7756	411
hypothetical protein	6246	7337	1092
hypothetical protein	4920	6185	1266
hypothetical protein	3908	4882	975
putative internal capsid protein	2604	3899	1296
hypothetical protein	2096	2611	516
hypothetical protein	1209	2084	876
putative capsid and scaffold protein	1	1209	1209

Table S3: Production of Interleukin 6

	Treatment	n	Minimum	25th Percentile	Median	75th percentile	Maximum
1	PBS (WT)	24	0.004683	0.081714	0.321699	0.863936	1.508449
2	Purified KPN7 (WT)	12	0.053838	0.247007	0.346057	0.530594	4.25139
3	Purified KPN8 (WT)	11	0.008165	0.100662	0.231325	0.652846	1.444081
4	LPS (WT)	48	318.0439	356.5376	388.132	443.0246	563.3722
5	Crude KPN7 (WT)	12	326.6528	370.7319	410.9947	456.1789	586.4728
6	Crude KPN8 (WT)	12	302.2242	344.0396	388.6336	438.9652	576.2055
7	PBS (IRAK3 Knockdown)	36	0.023252	0.064068	0.740428	0.821181	2.302732
8	Purified KPN7 (IRAK3 Knockdown)	6	0.001186	0.007536	0.044238	0.090391	0.167574
9	Purified KPN8 (IRAK3 Knockdown)	5	3.95E-05	0.001215	0.019774	0.029868	0.035626
10	LPS (IRAK3 Knockdown)	60	300.8543	442.9702	512.3772	667.7775	714.1284
11	Crude KPN7 (IRAK3 Knockdown)	6	600.918	677.143	698.4566	729.2409	735.9357
12	Crude KPN8 (IRAK3 Knockdown)	8	225.4606	347.4249	668.4639	704.1381	722.3294
13	PBS (IRAK3 Knockout)	28	0.42395	1.216423	1.93387	2.810114	2.810114
14	Purified KPN7 (IRAK3 Knockout)	6	0.001186	0.006875	1.096033	2.219724	2.286655
15	Purified KPN8 (IRAK3 Knockout)	7	0.00628	0.029254	0.05238	0.077644	0.188672
16	LPS (IRAK3 Knockout)	36	401.4144	556.7049	598.1317	652.4113	667.2087
17	Crude KPN7 (IRAK3 Knockout)	6	500.2721	619.7632	658.0342	698.0275	741.1082
18	Crude KPN8 (IRAK3 Knockout)	7	524.4332	553.0358	579.8908	671.0924	710.4177

Table S4: Production of Tumour Necrosis Factor – alpha

	Treatment	n	Minimum	25th percentile	Median	75th percentile	Maximum
1	PBS (WT)	8	0.001923	0.001923	0.018633	0.035343	0.035343
2	Purified KPN7 (WT)	8	0.004982	0.014155	0.023571	0.057764	0.168983
3	Purified KPN8 (WT)	6	0.847649	1.103309	1.438531	1.669659	1.787398
4	LPS (WT)	48	125.3497	131.2878	144.6694	154.3615	174.4363
5	Crude KPN7 (WT)	12	88.55341	93.10092	97.19952	108.1864	113.8395
6	Crude KPN8 (WT)	9	40.22049	41.3014	44.76724	46.45769	49.57528
7	PBS (IRAK3 Knockdown)	16	0.91773	0.9198	2.748813	4.651696	4.875372
8	Purified KPN7 (IRAK3 Knockdown)	4	0.174986	0.260158	0.928372	1.608419	1.729091
9	Purified KPN8 (IRAK3 Knockdown)	4	5.780675	5.867561	5.930849	5.977227	6.013384
10	LPS (IRAK3 Knockdown)	24	145.3778	148.5422	161.6254	166.9083	169.8639
11	Crude KPN7 (IRAK3 Knockdown)	6	251.2843	277.4774	291.9919	305.8648	331.2531
12	Crude KPN8 (IRAK3 Knockdown)	14	178.3251	225.0582	268.2506	394.5462	412.6336
13	PBS (IRAK3 Knockout)	16	5.832262	6.132885	6.654626	7.093746	7.146505
14	Purified KPN7 (IRAK3 Knockout)	4	5.700684	5.797821	11.37101	16.94528	17.04569
15	Purified KPN8 (IRAK3 Knockout)	6	1.505104	2.40468	4.560822	5.776204	6.438981
16	LPS (IRAK3 Knockout)	24	414.1599	422.6707	432.1394	438.7443	447.9709
17	Crude KPN7 (IRAK3 Knockout)	6	170.8852	188.566	192.3809	212.4994	228.836
18	Crude KPN8 (IRAK3 Knockout)	9	301.4171	305.905	382.9372	389.6463	390.2127
